# Renal Disorders with Oral Tyrosine Kinase Inhibitors in Metastatic Colorectal Cancer: An Analysis from the FDA Adverse Event Reporting System Database

**DOI:** 10.3390/biomedicines11082311

**Published:** 2023-08-20

**Authors:** Giulia Russo, Maria Antonietta Barbieri, Emanuela Elisa Sorbara, Giuseppe Cicala, Tindara Franchina, Mariacarmela Santarpia, Nicola Silvestris, Edoardo Spina

**Affiliations:** 1 Department of Clinical and Experimental Medicine, University of Messina, 98125 Messina, Italy; giuliarusso.ab@gmail.com (G.R.); mbarbieri@unime.it (M.A.B.); emanuela.sorbara@hotmail.it (E.E.S.); gcicala@unime.it (G.C.); 2 Department of Human Pathology in Adulthood and Childhood Gaetano Barresi, University of Messina, 98125 Messina, Italy; tfranchina@unime.it (T.F.); msantarpia@unime.it (M.S.); nsilvestris@unime.it (N.S.)

**Keywords:** colorectal cancer, pharmacovigilance, tyrosine kinase inhibitors, regorafenib, encorafenib, adverse drug reactions, renal disorders

## Abstract

Background: this study assessed the nephrotoxicity of regorafenib (REG) and encorafenib (ENC) in metastatic colorectal cancer (mCRC) through an analysis of reports from the US Food and Drug Administration’s Adverse Event Reporting System (FAERS) database. Methods: descriptive and disproportional analyses were performed for all reports using ENC and REG as the primary suspect. Results: A total of 379 reports had at least one renal adverse drug reaction (ADR), and these ADRs were mainly related to REG (93.1%). Potential safety signals for REG included chromaturia (*n* = 44; ROR = 12.00, CI 95% = 8.92–16.16; IC = 2.36, IC_025_–IC_075_ = 2.06–2.66), hydronephrosis (10; 8.70, 4.67–16.19; 1.85, 1.23–2.47), nephrotic syndrome (7; 5.73, 2.73–12.03; 1.47, 0.73–2.21), renal impairment (53; 4.16, 3.17–5.45; 1.39, 1.12–1.66), dysuria (19; 3.06, 1.95–4.81; 1.06, 0.61–1.52), renal failure (38; 1.66, 1.20–2.28; 0.49, 0.17–0.81), and acute kidney injury (AKI) (43; 1.46, 1.08–1.97; 0.37, 0.07–0.67). For ENC, consistent disproportionalities were observed for AKI (*n* = 11; ROR = 3.79, CI 95% = 2.09–6.90; IC = 1.32, IC_025_–IC_075_ = 0.72–1.91) and dysuria (4; 6.50, 2.43–17.39; 1.86, 0.88–2.85). Conclusions: these findings highlight some not extensively reported renal ADRs that require further investigations to better characterize the safety profiles of REG and ENC in patients with mCRC.

## 1. Introduction

Colorectal cancer (CRC) is considered to be the third most common cancer after lung and breast carcinoma, as well as the second leading cause of death worldwide after lung cancer. In the United States (US) alone, more than 150,000 new cases and over 52,000 related deaths were reported in 2022 [1,2]. Around 20% of new diagnoses confirm metastatic CRC (mCRC), while approximately 40% reveal localized cancer that later develops metastases [3,4]. From a molecular pathophysiology standpoint, CRC is characterized by specific molecular and mutational alterations. Approximately 40% of CRC patients have KRAS mutations, while 6% have NRAS mutations [5,6]. These mutations lead to the constitutive activation of the Ras-Raf-mitogen-activated protein kinase (MAPK) signaling pathway downstream of the epidermal growth factor receptor (EGFR). As a result, CRC exhibits resistance to anti-EGFR therapies, making the MAPK pathway the primary target [7,8]. Notably, mutations in the BRAF gene, which is a potent modulator of the MAPK pathway, are observed in approximately 8–12% of CRC patients, with BRAF-V600E accounting for over 95% of these mutations [9,10].

The identification of BRAF mutations has led to the development of specific oral targeted agents, which are currently used for the treatment of mCRC patients harboring these alterations. These therapies offer several advantages over injectable formulations, including flexibility, convenience, cost-effectiveness (in some cases), and improved compliance [11,12]. Currently, two types of oral tyrosine kinase inhibitors (TKIs) are approved for the treatment of mCRC: regorafenib (REG), which has been available since 2012, is identified as a multikinase inhibitor, since it inhibits antigenic and oncogenic kinases, such as vascular endothelial growth factor receptors (VEGFR), platelet-derived growth factor receptors (PDGFR), fibroblast growth factor receptors (FGFR), and BRAF; and encorafenib (ENC), which is a pure BRAF inhibitor (BRAFi) approved for use in combination with cetuximab (CET) for the treatment of mCRC in 2020 [13,14].

Although the introduction of REG and ENC has improved survival in patients with mCRC, their use is not exempt from adverse events (AEs). In pre-marketing studies, almost 91% of patients treated with REG experienced at least one AE, including serious ones, with fatigue (46%), hand–foot skin reaction (HFSR) (42%), and hypertension (30%) being the most common AEs [15]. Gastrointestinal disorders are primarily reported for ENC, along with fatigue (33%) and dermatitis acneiform (30%) [16]. Due to the clinical relevance of some AEs that have not yet been fully characterized, it may be beneficial to use real-word data from the spontaneous reporting system (SRS) databases. Renal adverse drug reactions (ADRs) have been identified in several studies, suggesting that nephrotoxicity may be a common class effect of TKIs, including BRAFi [16,17,18]. However, the specific mechanisms underlying kidney-related ADRs are not fully understood. Additionally, only proteinuria is reported to be a renal and urinary disorder in the label of REG [13].

Currently, only a few studies have been conducted for REG [19,20,21], and there are no studies that directly compare the safety profiles of REG and ENC plus CET. The SRS databases play crucial roles in identifying new ADRs and may, therefore, contribute to improving the quality of life (QoL) and overall clinical outcomes of treatment. For this reason, the aim of the study was to evaluate and characterize the safety profiles of oral TKIs, REG, and ENC for mCRC, with a specific focus on renal and urinary disorders. This was achieved by conducting an analysis of the US Food and Drug Administration’s Adverse Event Reporting System (FAERS).

## 2. Materials and Methods

### 2.1. Data Source and Case Definition

An observational retrospective pharmacovigilance study was conducted based on reports of suspected ADRs collected via the FAERS database. FAERS is one of the most widely used publicly available databases, gathering more than 20 million reports from patients, healthcare professionals, and pharmaceutical companies in the USA, Europe, and Asia. The reported information includes identification numbers, dates of receipts and events, the reporting country, qualifications of primary sources, patient characteristics (e.g., gender, age, and weight), and suspected and concomitant drugs and their indications, ADRs, and seriousness of ADRs. All ADRs are coded using the Medical Dictionary for Regulatory Activities (MedDRA^®^) maintained by the Maintenance and Support Services Organization (MSSO), McLean, VA, USA [22] in terms of the involved organ or system and corresponding signs/symptoms, which were referred to as System Organ Class (SOC) and Preferred Term (PT), respectively.

All of the zipped ASCII FAERS quarterly data extract files from Q4 2012 to Q4 2022 (https://fis.fda.gov/extensions/FPD-QDE-FAERS/FPD-QDE-FAERS.html (accessed on 30 January 2023)) were downloaded and processed to remove duplicates. Duplicate reports were identified based on overlapping information in key fields, including AE, event date, gender, age, body weight, reporting country, and suspected active substances. Reports with a unique Case ID related to REG and ENC (primary suspect), with CRC as the indication, collected from October 2012 (the date of REG’s first approval for the treatment of mCRC) to December 2022 were included.

Reports that contained at least one ADR belonging to the SOC “Renal and urinary disorders” were selected as “cases”, while the remaining reports were identified as “non-cases”. The selection process at the case level was based on the identification number, ensuring that reports with multiple renal ADRs were only counted once.

### 2.2. Data Analyses

A descriptive statistical analysis was conducted to assess the demographic and clinical characteristics of the FAERS reports related to renal disorders. Specifically, patient characteristics (gender and age), primary source of information, the year of reporting, the reporting country, and ADR characteristics, including seriousness, outcome, and time to onset (TTO), were described. The TTO is based on the period between drug administration and the onset of the ADR, and it was expressed as a median with interquartile range (Q1–Q3). Continuous variables were presented as medians (Q1–Q3), while categorical variables were reported as absolute (percentage) values. To compare the reports with at least one renal ADR to all other FAERS reports, differences in categorical variables were evaluated using Pearson’s chi-square test on a 2 × 2 contingency table with Yates’ continuity correction. Differences in continuous variables were assessed using the Mann–Whitney U test.

An exploratory disproportionality analysis using a case/non-case methodology was performed to identify new and previously undetected renal ADRs via PTs. The reporting odds ratio (ROR) with the corresponding 95% confidence interval (CI) was calculated to perform this analysis. The statistical baseline was determined based on the lower limit of the 95% of the ROR being > 1, with a minimum of 3 reports for each drug-event pair. ADRs that were not reported in the FDA Full Prescribing Information at the time of the study were considered to be unexpected. To minimize the risk of detecting false associations and evaluate the strength of the association between REG or ENC and the onset of renal ADRs, the Bayesian information component (IC) was calculated. The lower limit of a 95% credibility interval > 0 (IC_025_ > 0) was indicative of a connection between the drug and the ADR [23,24].

A statistically significant threshold was set at a *p* value of <0.05 for all analyses. All statistical analyses were performed using SPSS version 23.0 (IBM Corp., SPSS Statistics, Armonk, NY, USA).

## 3. Results

### 3.1. Descriptive Analysis

**Figure 1:**
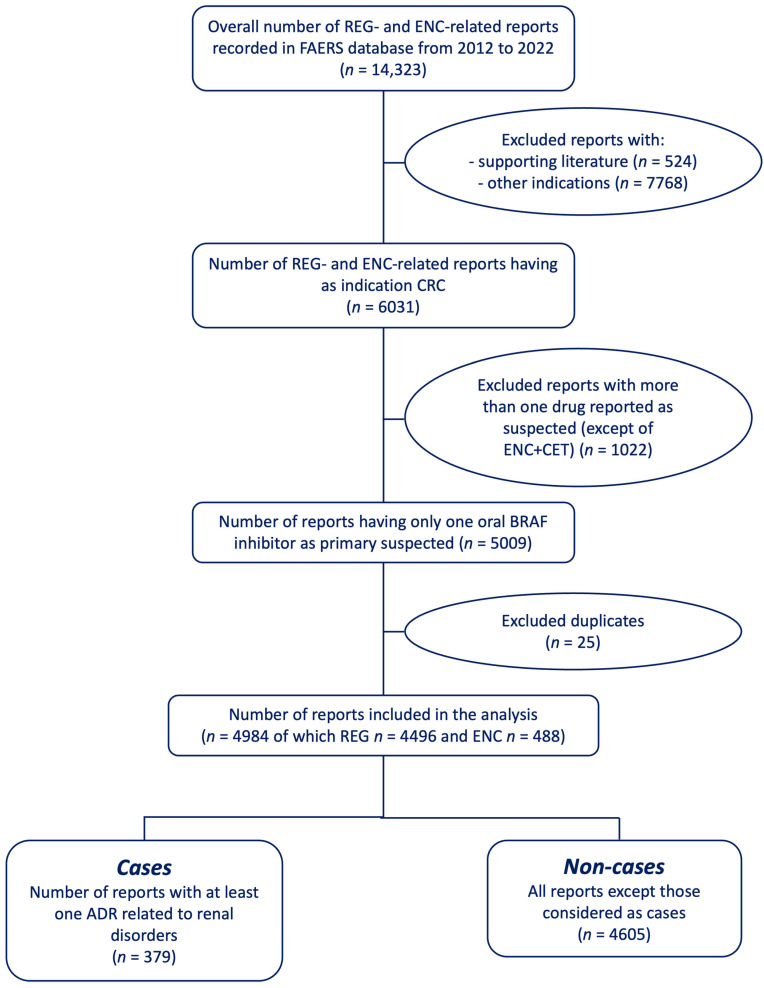
Flowchart of the reports selection process. Abbreviations: FAERS = US Food and Drug Administration’s Adverse Event Reporting System (FAERS) database; mCRC = metastatic colorectal cancer; REG = regorafenib; ENC = encorafenib; CET = cetuximab.

From October 2012 to December 2022, a total of 14,323 reports referring to REG and ENC were recorded in the FAERS database. After excluding pre-marketing reports supported by the literature (*n* = 524), reports related to other indications (*n* = 7768), reports related to another drug identified as the primary suspect (*n* = 1022), and duplicates (*n* = 25), a total of 4984 reports were included in the analysis. Among these reports, a majority were related to REG (*n* = 4496; 90.2%), followed by ENC (*n* = 488; 9.8%). Among the reports in which ENC was the primary suspect, 150 cases indicated that CET was a secondary suspected drug (30.7%). There were 379 (7.6%) reports containing at least one renal ADR (cases) (Figure 1).

Renal ADR reports were more frequently related to males compared to all other reports (60.7% vs. 51.8%, *p* = 0.006). However, there was no statistically significant difference in the distribution of cases between the adult and elderly populations. Consumers were the primary reporters of cases compared to non-cases (48.5% vs. 42.1%, *p* = 0.025), particularly in the years 2016 and 2018 (10.8% vs. 6.9%, *p* = 0.006 and 13.2% vs. 9.0%, *p* = 0.010, respectively). Renal ADRs were more likely to be classified as serious compared to all other reports (97.6% vs. 89.5%, *p* < 0.001) and had a higher frequency of hospitalization and life-threatening outcomes (39.6% vs. 27.6%, *p* < 0.001 and 4.0% vs. 2.1%, *p* = 0.028, respectively). REG was predominantly involved in renal ADRs compared to all other reports (93.1% vs. 90.0%, *p* = 0.046) (Table 1).

**Figure 2:**
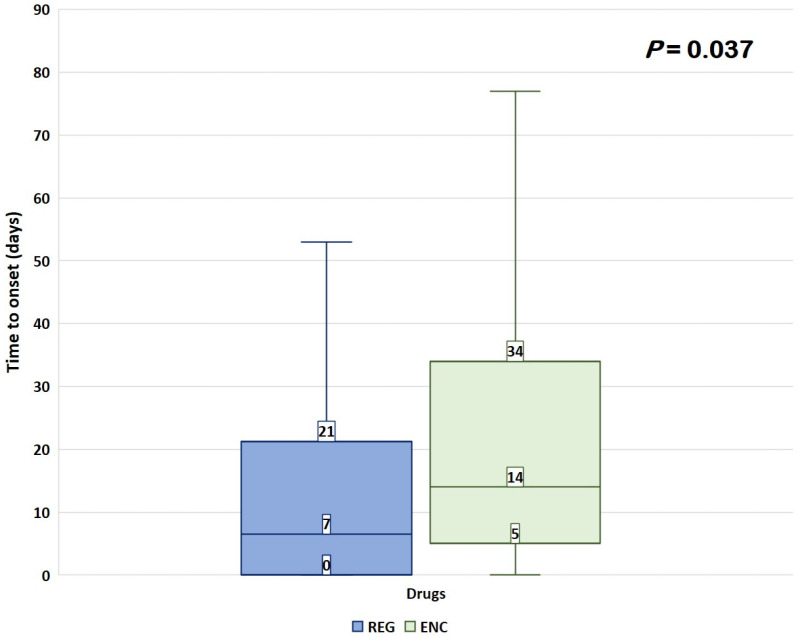
Time to onset of renal ADRs. Data are reported as box plots, with the box drawn from Q1 to Q3 and the horizontal line drawn in the middle to denote the median. Abbreviations: REG = regorafenib; ENC = encorafenib.

The median (Q1–Q3) TTO of renal ADRs was higher with ENC than with REG [14 (5–34) vs. 7 (0–21) days, *p* = 0.037] (Figure 2).

Renal and urinary disorders primarily involved renal impairment (*n* = 56; 14.8%), followed by acute kidney injury (AKI) (*n* = 54; 14.3%), chromaturia (*n* = 44; 11.6%), and renal failure (*n* = 40; 10.6%). Among these disorders, renal impairment, chromaturia, and renal failure were mostly reported for REG (in order *n* = 53; 15.0%, *n* = 44; 12.7%, and *n* = 38; 10.8%), while AKI was more commonly associated with ENC (*n* = 11; 42.3%).

### 3.2. Disproportionality Analysis

Relevant disproportionality signals of ADRs related to REG and belonging to the SOC renal and urinary disorders were already known and mentioned in the FDA Full Prescribing Information, including proteinuria, haematuria, and haemorrhage of the urinary tract. However, this analysis also revealed consistent new potential safety signals for REG, such as prerenal failure (*n* = 4; ROR = 22.13, CI 95% = 8.29–59.11; IC = 1.89, IC_025_–IC_075_ = 0.91–2.87), chromaturia (44; 12.00, 8.92–16.16; 2.36, 2.06–2.66), urinary tract obstruction (5; 9.16, 3.81–22.04; 1.66, 0.78–2.54), hydronephrosis (10; 8.70, 4.67–16.19; 1.85, 1.23–2.47), micturition disorder (3; 5.78, 1.86–17.95; 1.23, 0.10–2.37), nephrotic syndrome (7; 5.73, 2.73–12.03; 1.47, 0.73–2.21), renal pain (6; 4.08, 1.83–9.10; 1.19, 0.39–1.99), urinary retention (27; 4.99, 3.42–7.28; 1.53, 1.15–1.91), urine odour abnormal (4; 4.61, 1.73–12.28; 1.19, 0.21–2.17), renal impairment (53; 4.16, 3.17–5.45; 1.39, 1.12–1.66), anuria (6; 3.48, 1.56–7.76; 1.07, 0.27–1.87), nocturia (6; 3.39, 1.52–7.55; 1.05, 0.25–1.85), oliguria (5; 3.25, 1.35–7.81; 0.99, 0.11–1.87), dysuria (19; 3.06, 1.95–4.81; 1.06, 0.61–1.52), renal disorder (19; 2.72, 1.73–4.26; 0.95, 0.50–1.40), urinary incontinence (13; 2.46, 1.43–4.24; 0.85, 0.30–1.39), renal failure (38; 1.66, 1.20–2.28; 0.49, 0.17–0.81), and AKI (43; 1.46, 1.08–1.97; 0.37, 0.07–0.67) (Table 2).

For ENC, the analysis showed consistent disproportionality signals for renal ADRs that were not reported in the FDA Full Prescribing Information, such as AKI (*n* = 11; ROR = 3.79, CI 95% = 2.09–6.90; IC = 1.32, IC_025_–IC_075_ = 0.72–1.91) and dysuria (4; 6.50, 2.43–17.39; 1.86, 0.88–2.85) (Table 2).

## 4. Discussion

This study, which focuses on renal disorders associated with REG and ENC in patients with mCRC using the FAERS database, can be considered to be the first of its kind. Renal disorders accounted for approximately 8% of all of the reports analyzed. The findings of this analysis revealed a higher association between renal ADRs and males, with an equal distribution between the adult and elderly population, compared to other cases. Previous studies have indicated that men may have a higher risk of developing renal impairment with targeted therapies than women [25,26]. This observation could be attributed to testosterone, which has been implicated in the progression of chronic kidney disease (CKD) and the worsening of kidney function in men [27]. The onset of renal ADRs in both adult and elderly patients could be related to the early screening of mCRC in adults [2], as well as the presence of comorbidities commonly found in elderly patients diagnosed with cancer, such as hypertension or diabetes mellitus, along with pre-existing kidney dysfunction. These factors can substantially contribute to the development of nephrotoxicity [28]. Notably, two case reports have confirmed instances of renal impairment in two patients aged 62 and 70, respectively, who received REG treatment for mCRC [29,30].

Renal ADRs were predominantly classified as serious compared to other reported ADRs, as evidenced by the literature data on TKIs, in which a significant proportion (79%) of renal reports were serious [26]. In contrast to the previous pharmacovigilance study, a clinical trial reported serious renal disorders of grade 3 in only 10% of patients [31]. This finding suggests that the occurrence of severe renal complications associated with the treatment may vary between real-life studies and clinical trials, underscoring the importance of pharmacovigilance in safety investigations. The higher frequency of renal ADRs requiring hospitalization can be explained by the complexity of patients, particularly older adults, who often receive polytherapy and may have multi-organ damage that poses a risk to their own life [32,33].

The median TTO for renal ADRs was found to be higher with ENC than REG. In the literature, the median TTOs for all TKIs varied significantly, ranging from 26 days to 684 days for renal disorders [26,34]. This greater difference could be attributed to the fact that different TKIs, including ENC and REG, may have distinct renal effects and toxicity profiles, leading to variations in the occurrence of serious renal disorders. However, a previous study reported a similar median TTO of 27 days for AKI in patients treated with ENC, which aligns with our findings [35]. Furthermore, three case series have indicated the presence of two types of kidney injury associated with BRAFi. One type manifested shortly after the initiation of drug treatment, typically within 1–2 weeks. The other type of kidney injury had a more gradual onset and became apparent within 1–2 months [18].

Despite the extensive explanations reported in the literature regarding the occurrence of renal disorders with REG and ENC, several relevant disproportionality signals related to REG and ENC in the SOC renal and urinary disorders have not been mentioned in the FDA Full Prescribing Information. One possible explanation is that REG functions as a multikinase inhibitor, affecting molecules, such as the vascular–endothelial growth factor (VEGF), that play a crucial role in the glomerular filtration barrier. This issue can result in increased proteinuria and the development of thrombotic microangiopathy (TMA) [29,36,37]. Additionally, REG, like other well-documented anti-angiogenetic agents, may exhibit dose-dependent nephrotoxicity [38]. Moreover, renal impairment has been associated with BRAFi, including ENC. This finding aligns with kidney biopsy results observed in patients treated with other BRAFi, such as vemurafenib, which showed signs of acute and chronic tubular injury [39]. However, the underlying mechanisms of nephrotoxicity in relation to BRAFi are not yet fully understood. One possible mechanism is the interaction between BRAFi and tubular creatinine secretion, which may induce acute tubular necrosis (ATN) [40]. Furthermore, the potential involvement of ENC in association with CET cannot exclude the role of CET itself in the development of renal disorders. This issue can be explained by the role of EGFR in cell regeneration following ATN. The use of anti-EGFR therapies, such as CET, may potentially hinder the re-epithelialization of tubules and impede the recovery process [41]. Additionally, in the European Medicines Agency (EMA)’s Summary of Product Characteristics (SmPC), renal failure is reported to be an ADR associated with ENC [42].

Considering all of the disproportionality analyses, potential safety signals were identified for REG, including pre-renal failure, renal failure, and AKI. These ADRs could also be associated with other potential safety signals, such as renal impairment, renal disorders, and renal pain. Pre-renal failure and AKI typically occur as a result of extrarenal diseases that lead to a decrease in the glomerular filtration rate [43]. TKIs may induce AKI through two mechanisms: toxic injury to the renal tubules and the occurrence of tumor lysis syndrome [44]. Furthermore, TKIs have the potential to cause injury to podocytes by inducing the tyrosine phosphorylation of nephrin, which is a critical protein in maintaining the integrity of the filtration barrier. The loss of normal podocyte fenestration can lead to various complications, including microvascular injury, capillary thrombosis, and the development of renal glomeruli sclerotic lesions, ultimately resulting in renal failure [45].

Regarding the potential signal related to nephrotic syndrome, a previous study found that the known side effect of REG, i.e., proteinuria, could lead to minimal change nephrotic syndrome and TMA [46]. Interestingly, TMA has been found to occur more frequently in anti-VEGF therapies, such as bevacizumab, while nephrotic syndrome has been associated with other TKIs, including dasatinib [47]. It is worth noting that instances of nephrotic syndrome, with or without TMA characteristics, noted as severe side effects of TKIs in adult cancer patients, have been infrequently reported in the literature [30,48].

The disproportionality analysis revealed a significant association between REG and various urinary ADRs, including micturition disorder, urinary retention, urinary tract obstruction, urinary incontinence, anuria, nocturia, dysuria, oliguria, hydronephrosis, chromaturia, and urine odour abnormal. Furthermore, it is important to note that urinary tract obstruction and hydronephrosis could potentially be complications or exacerbations of the mCRC condition. Therefore, reports containing these ADRs should be approached with careful consideration. In the REG FDA Full Prescribing Information, burning or painful urination is reported to be a symptom associated with infections [13]. It is possible that the presence of an infection itself can lead to difficulties or discomfort during urination, which encompasses all of the potential signals mentioned above. Furthermore, urinary disorders, including chromaturia, may be associated with other ADRs, such as severe bleeding and liver problems. According to the FDA Full Prescribing Information, pink or brown urine may indicate severe bleeding, while dark “tea-colored” urine may suggest liver problems [13]. Additionally, an abnormal urine odour could potentially indicate the development of CRC. Unusual changes in the smell of urine may serve as a notable symptom that should prompt further investigation or medical evaluation to assess the possibility of underlying CRC [49]. Moreover, dysuria was identified as a potential safety signal for ENC. A previous pre-marketing study demonstrated the occurrence of urinary tract infection in approximately 8% of patients receiving ENC and CET [16]. Urinary tract infection is one of the most common causes of dysuria [50]. Therefore, the onset of dysuria could be a consequence of the onset of this infection.

### Strengths and Limitations

The risk/benefit profile of oral TKIs for mCRC appears to be well characterized. However, renal ADRs are not fully mentioned in the FDA Full Prescribing Information for REG and ENC. The strength of this study lies in the large number of reports analyzed, which contribute to the cumulative knowledge of the nephrotoxicity of oral TKIs in mCRC. The use of a global database and the combination of a disproportionality approach with case/non-case evaluation has been documented in the literature [23,51]. One of the main advantages of using the SRS database is its ability to generate new potential safety signals for ADRs that may remain undetected during the pre-marketing phase [52]. Patients with cancer often experience a lower health-related QoL, which can be influenced by the use of chemotherapeutic agents, including second- and third-line therapies, as previously observed [53,54]. Notably, the increased use of TKIs as a second-line therapy in patients with mCRC following prior treatment with nephrotoxic chemotherapeutic agents may impact QoL [28,38,55,56]. Moreover, renal disorders can worsen over the duration of the tumor course and the progression of metastases. In this context, it would be interesting to analyze, in a real-world setting, whether the patterns of metastatic disease in CRC (e.g., bone metastasis, which can be associated with hypercalcemia and hypercalciuria) could influence the development of renal ADRs. Therefore, the timely detection of ADRs can assist oncologists in determining the best treatment choices for patients affected by mCRC.

However, the FAERS database may not always provide comprehensive information about potential confounding factors. Details such as a patient’s past medical history, concomitant treatments, and precise dosing and frequency of drug administration may not always be available, which limits a doctor’s ability to fully assess the impact of these factors on kidney toxicity. Various factors, including gender differences [27] and underlying conditions associated with mCRC, such as diarrhea, dehydration, bone marrow suppression, and infections, could also contribute to kidney toxicity [34]. The absence of drug users as a denominator, under-reporting or over-reporting phenomena, and the lack of specific data in the FAERS database can pose challenges to establishing a clear causal relationship between the use of oral TKIs and the occurrence of renal ADRs. Although disproportionality analysis is a validated method used in drug safety research and surveillance to identify potential signals of ADRs, it is crucial to acknowledge that disproportionality analysis alone should be considered to be an exploratory approach that generates signals, rather than providing definitive confirmation of causality [57].

## 5. Conclusions

In recent years, the use of oral TKIs as a second-line treatment for mCRC has brought improvements in medical treatment and patient compliance. The case/non-case analysis has highlighted some ADRs that have not been extensively reported in the literature but are worth discussing, such as AKI, renal failure, nephrotic syndrome, and urinary conditions associated with REG and ENC. These renal ADRs can have significant impacts on each patient’s QoL and treatment outcome.

Encouraging collaboration and mutual learning between oncologists and nephrologists is essential to improving patient care, managing clinical symptoms, and minimizing the onset of nephrotoxicity with oral TKIs in mCRC patients. It is crucial to expand the knowledge of renal ADRs and their impact on the well-being of patients affected by mCRC through further pharmacovigilance studies. Such studies will contribute to a better understanding of nephrotoxicity and enable the development of strategies to enhance patient well-being and treatment management.

## Figures and Tables

**Table 1:** Characteristics of reports with renal disorders related to regorafenib or encorafenib.

Characteristic	Renal Cases (*n* = 379)	Other Reports (*n* = 4605)	*p* Value	Total (*n* = 4984)
Gender, *n* (%)				
Male	230 (60.7)	2384 (51.8)	**0.006**	2614 (52.4)
Female	141 (37.2)	1991 (43.2)		2132 (42.8)
Not specified	8 (2.1)	230 (5.0)		238 (4.8)
Median age (Q1–Q3), years	65 (55–71)	64 (56–71)	0.886	64 (56–71)
Age group, *n* (%)				
Adult	170 (44.9)	1990 (43.3)	0.494 *	2160 (43.3)
18–29 years	0 (0.0)	26 (0.6)	0.197	26 (0.5)
30–49 years	40 (10.6)	399 (8.7)		439 (8.8)
50–64 years	130 (34.3)	1565 (34.0)		1695 (34.0)
Elderly	170 (44.9)	1830 (39.8)		2000 (40.1)
65–75 years	125 (33.0)	1283 (27.9)	0.449	1408 (28.3)
76–85 years	42 (11.1)	487 (10.6)		529 (10.6)
>85 years	3 (0.8)	60 (1.3)		63 (1.3)
Missing	39 (10.3)	785 (17.0)		824 (16.5)
Reporter type, *n* (%)				
Consumer	184 (48.5)	1941 (42.1)	**0.025**	2125 (42.6)
Healthcare professional	194 (51.2)	2617 (56.8)		2811 (56.4)
Not specified	1 (0.3)	47 (1.0)		48 (1.0)
Reporter Country, *n* (%)				
Africa	1 (0.3)	34 (0.7)	0.457	35 (0.7)
Asia	98 (25.9)	1063 (23.1)	0.244	1161 (23.3)
Europe	75 (19.8)	783 (17.0)	0.190	858 (17.2)
North America	177 (46.7)	2199 (47.8)	0.734	2376 (47.7)
Oceania	4 (1.1)	49 (1.1)	0.987	53 (1.1)
South America	12 (3.2)	141 (3.1)	0.910	153 (3.1)
Not specified	12 (3.2)	336 (7.3)	-	348 (7.0)
Serious, *n* (%)	370 (97.6)	4122 (89.5)	**<0.001**	4492 (90.1)
Outcome, *n* (%)				
Died	78 (20.6)	970 (21.1)	0.876	1048 (21.0)
Disabled	5 (1.3)	53 (1.2)	0.964	58 (1.2)
Hospitalized	150 (39.6)	1273 (27.6)	**<0.001**	1423 (28.6)
Life threatening	15 (4.0)	96 (2.1)	**0.028**	111 (2.2)
Non-serious	9 (2.4)	483 (10.5)	**<0.001**	492 (9.9)
Other outcomes	122 (32.2)	1727 (37.5)	**0.045**	1849 (37.1)
Required intervention	0 (0.0)	3 (0.1)	-	3 (0.1)
Year of reporting, *n* (%)				
2012	6 (1.6)	75 (1.6)	0.946	81 (1.6)
2013	40 (10.6)	619 (13.4)	0.129	659 (13.2)
2014	33 (8.7)	477 (10.4)	0.352	510 (10.2)
2015	47 (12.4)	491 (10.7)	0.336	538 (10.8)
2016	41 (10.8)	316 (6.9)	**0.006**	537 (7.2)
2017	38 (10.0)	437 (9.5)	0.802	475 (9.5)
2018	50 (13.2)	416 (9.0)	**0.010**	466 (9.3)
2019	34 (9.0)	366 (7.9)	0.544	400 (8.0)
2020	25 (6.6)	458 (9.9)	**0.043**	483 (9.7)
2021	34 (9.0)	515 (11.2)	0.216	549 (11.0)
2022	31 (8.2)	435 (9.4)	0.470	466 (9.3)
Primary suspect drug				
ENC	26 (6.9)	462 (10.0)	**0.046**	488 (9.8)
REG	353 (93.1)	4143 (90.0)		4496 (90.2)

Significant *p* values are shown in bold type. ENC = encorafenib; Q1 = quartile 1; Q3 = quartile 3; REG = regorafenib. * Calculated as adults vs. elderly.

**Table 2:** Disproportionality analyses, included ROR and IC, and notoriety based on FDA label for renal and urinary ADRs related to ENC and REG.

Preferred Term	ENC				REG				Total
	*n*	ROR (95% CI)	IC (IC_025_–IC_075_)	Unexpected	*n*	ROR (95% CI)	IC (IC_025_–IC_075_)	Unexpected	
Renal impairment	3	2.35 (0.75–7.31)			53	**4.16 (3.17–5.45)**	1.39 (1.12–1.66)	Yes	56
AKI	11	**3.79 (2.09–6.90)**	1.32 (0.72–1.91)	Yes	43	**1.46 (1.08–1.97)**	0.37 (0.07–0.67)	Yes	54
Chromaturia					44	**12.00 (8.92–16.16)**	2.36 (2.06–2.66)	Yes	44
Renal failure	2	NA			38	**1.66 (1.20–2.28)**	0.49 (0.17–0.81)	Yes	40
Proteinuria					29	**11.01 (7.64–15.86)**	2.24 (1.87–2.60)	No	29
Urinary retention					27	**4.99 (3.42–7.28)**	1.53 (1.15–1.91)	Yes	27
Dysuria	4	**6.50 (2.43–17.39)**	1.86 (0.88–2.85)	Yes	19	**3.06 (1.95–4.81)**	1.06 (0.61–1.52)	Yes	23
Haematuria					22	**3.29 (2.16–5.00)**	1.14 (0.72–1.56)	No	22
Renal disorder	1	NA			19	**2.72 (1.73–4.26)**	0.95 (0.50–1.40)	Yes	20
Urinary incontinence					13	**2.46 (1.43–4.24)**	0.85 (0.30–1.39)	Yes	13
Hydronephrosis	1	NA			10	**8.70 (4.67–16.19)**	1.85 (1.23–2.47)	Yes	11
Pollakiuria	2	NA			8	1.18 (0.59–2.36)			10
Renal pain	2	NA			6	**4.08 (1.83–9.10)**	1.19 (0.39–1.99)	No	8
Nephrolithiasis	1	NA			6	0.95 (0.43–2.11)			7
Nephrotic syndrome					7	**5.73 (2.73–12.03)**	1.47 (0.73–2.21)	No	7
Anuria					6	**3.48 (1.56–7.76)**	1.07 (0.27–1.87)	Yes	6
Nocturia					6	**3.39 (1.52–7.55)**	1.05 (0.25–1.85)	Yes	6
Oliguria					5	**3.25 (1.35–7.81)**	0.99 (0.11–1.87)	Yes	5
Urinary tract obstruction					5	**9.16 (3.81–22.04)**	1.66 (0.78–2.54)	Yes	5
Haemorrhage urinary tract					4	**11.23 (4.21–29.96)**	1.66 (0.68–2.64)	No	4
Micturition urgency					4	2.28 (0.86–6.08)			4
Prerenal failure					4	**22.13 (8.29–59.11)**	1.89 (0.91–2.87)	Yes	4
Urine odour abnormal					4	**4.61 (1.73–12.28)**	1.19 (0.21–2.17)	Yes	4
Bladder disorder					3	1.96 (0.63–6.09)			3
CKD					3	0.23 (0.07–0.72)			3
Micturition disorder					3	**5.78 (1.86–17.95)**	1.23 (0.10–2.37)	Yes	3

Significant RORs are shown in bold type. NA = not available because there were fewer than three reports; AKI = acute kidney injury; CI = confidence interval; CKD = chronic kidney disease; ENC = encorafenib; NA = not applicable; IC = information component; PT = preferred term; REG = regorafenib; ROR = reporting odds ratio.

## Data Availability

The datasets analyzed during the current study are available using the following resource that is available in the public domain: https://fis.fda.gov/extensions/FPD-QDE-FAERS/FPD-QDE-FAERS.html (accessed on 30 January 2023).
